# How Cells Communicate with Each Other in the Tumor Microenvironment: Suggestions to Design Novel Therapeutic Strategies in Cancer Disease

**DOI:** 10.3390/ijms22052550

**Published:** 2021-03-04

**Authors:** Roberto Zefferino, Claudia Piccoli, Sante Di Gioia, Nazzareno Capitanio, Massimo Conese

**Affiliations:** 1 Department of Medical and Surgical Sciences, University of Foggia, 71122 Foggia, Italy; sante.digioia@unifg.it (S.D.G.); massimo.conese@unifg.it (M.C.); 2 Department of Clinical and Experimental Medicine, University of Foggia, 71122 Foggia, Italy; claudia.piccoli@unifg.it (C.P.); nazzareno.capitanio@unifg.it (N.C.)

**Keywords:** connexin, pannexin, hemichannels, gap junction intercellular communication, tumor microenvironment, epithelial-mesenchymal transition, purinergic system, inflammasome, immune system, cytokines

## Abstract

Connexin- and pannexin (Panx)-formed hemichannels (HCs) and gap junctions (GJs) operate an interaction with the extracellular matrix and GJ intercellular communication (GJIC), and on account of this they are involved in cancer onset and progression towards invasiveness and metastatization. When we deal with cancer, it is not correct to omit the immune system, as well as neglecting its role in resisting or succumbing to formation and progression of incipient neoplasia until the formation of micrometastasis, nevertheless what really occurs in the tumor microenvironment (TME), which are the main players and which are the tumor or body allies, is still unclear. The goal of this article is to discuss how the pivotal players act, which can enhance or contrast cancer progression during two important process: “Activating Invasion and Metastasis” and the “Avoiding Immune Destruction”, with a particular emphasis on the interplay among GJIC, Panx-HCs, and the purinergic system in the TME without disregarding the inflammasome and cytokines thereof derived. In particular, the complex and contrasting roles of Panx1/P2X7R signalosome in tumor facilitation and/or inhibition is discussed in regard to the early/late phases of the carcinogenesis. Finally, considering this complex interplay in the TME between cancer cells, stromal cells, immune cells, and focusing on their means of communication, we should be capable of revealing harmful messages that help the cancer growth and transform them in body allies, thus designing novel therapeutic strategies to fight cancer in a personalized manner.

## 1. Introduction

Cancer is a multifactorial disease [1,2]. Cell–cell communication plays a fundamental role in maintaining tissue homeostasis and responding to both external and internal stimuli. In 1966, Loewenstein and Kanno, describing the inhibition of GJs (Gap Junctions) in cancer cells, hypothesized that GJIC (Gap Junctional Intercellular Communication) was involved in the early stages of carcinogenesis process [3], while Kar et al. [4] have later proposed that GJIC is crucial in the metastatic process. Regarding the immune system’s role in contrasting incipient neoplasia and in late-stage tumors, the micrometastasis formation is doubtless. The long-standing theory of immune surveillance suggests that the immune system uses an incessant early warning system capable of controlling cells and tissues that would recognize and eliminate the huge majority of incipient cancer cells and tissue nascent tumors. The goal of this article is to discuss how GJIC works during the “Activating Invasion and Metastasis” and how it acts during the “Avoiding Immune Destruction”, deepening two important stages of cancer progression.

**Figure 1:**
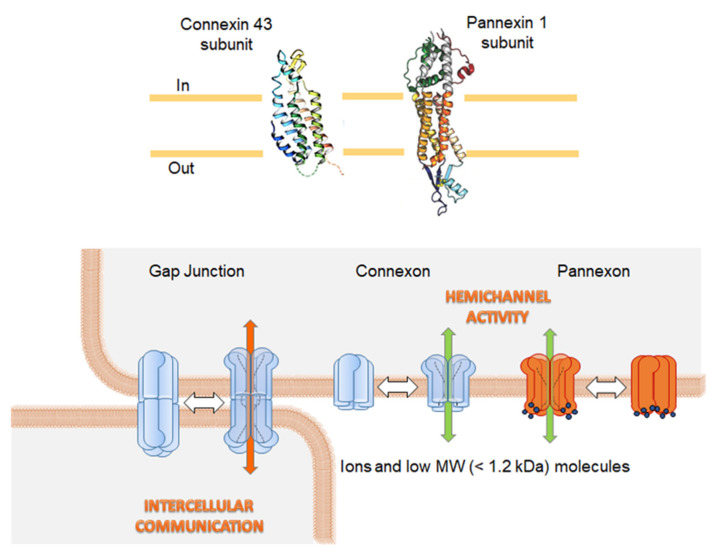
The assembly of connexins and pannexins into hemichannels and gap junctions. The upper panel shows the structure of connexin 43 and pannexin 1 subunits. The lower panel (on the right hand) illustrates how connexins and pannexins assemble into hexamers, called connexon and pannexon, respectively, to form a hemichannel. Docking of two hemichannels from two neighboring cells form a gap junction (on the left hand). They permit the cells to exchange ions and small low molecular weight (MW) molecules with <1.2 kDa, that is intercellular communication. The shown protein structures of connexin 43 and pannexin 1 were taken from the RCSB-protein data bank (http://www.rcsb.org/ access date: 31 January 21).

Intercellular communication plays an operative role in many processes, including organogenesis, homeostasis, regeneration processes, immune response, electrical coupling of excitable cells, and cancer [5,6]. In particular, gap junctions (GJs), channels allowing the passage of ions and small molecules (<~1.2 kDa) from one cell to a neighboring one, are involved not only in electrical coupling but also have a role in non-excitable cells concerning proliferation/differentiation and the maintenance of tissue homeostasis [7,8]. GJs are formed by protein family members called connexins (Cxs). Each Cx monomer is composed of four transmembrane domains, two extracellular loops, and cytoplasmic N and C termini (Figure 1). Cxs form hexamers, called connexons or hemichannels (HCs), homomeric or heteromeric if the aggregate is the same or different Cx isoforms, respectively (Figure 1). Connexin 43 (Cx43) is the connexin isoform more expressed in human tissues [9].

While Cxs are present in the chordate, invertebrates use innexins (Inxs) to form intercellular GJs [10]. However, Inxs genes are also present in vertebrates and non-vertebrates and were renamed as pannexins (Panxs), from the Latin prefix “pan,” meaning “all” [11]. Similar to Cxs, Panxs show a N-terminal domain and a C-terminal domain in the cytosol, four transmembrane domains, and two extracellular loops [12]. Because Panxs present high level of glycosylation in their extracellular domains, they are able to form HCs, or pannexons [13,14,15,16,17,18] (Figure 1).

It has been reported that there are three ways that Cxs use to modulate cellular processes [19]: The first considers the GJ Intercellular Communication (GJIC), and indeed through GJIC the cell may communicate with neighboring cells via small molecules and ions exchange. In the past, it was shown that this function is useful to control cell proliferation. Promoter carcinogens act as such by reducing GJIC. While the GJIC permits the cells to communicate to each other, the second process considered by Syniuk and colleagues [19] takes into account the enabled communication between cells and the extracellular milieu. The structures capable of connecting the cells with extracellular matrix (ECM) are the HCs, constituted by both Cxs and Panxs. If the molecules that cells exchange by GJIC are many, and a few perhaps not yet identified, the functional significance of HCs appears to be different, in that the role of ATP and then of the purinergic system seem to be hallmarking. The third mechanism reported is the interaction between Cxs and several proteins, at the level of the Cx C or N-terminals. Here the complexity of the problem increases, and further studies are needed to define the functional consequence of these interactions as several reported data appear contradictory.

Among the three proteins belonging to the pannexin family, Panx1 is the most ubiquitously expressed in mammalian tissues [20,21]. Although endogenously expressed, Panx1 localizes on the plasma membrane and its overexpression leads to the formation of Panx1 Ca^2+^-permeant channels at the level of endoplasmic reticulum [22], suggesting that Panx1 could contribute to sustained Ca^2+^ intracellular levels. Panxs form channels that are implicated in a regulated release of ATP, that, in turn is involved in different physiological functions: Not only cell proliferation, but also migration and differentiation [23,24], as well as in pathophysiological events characterizing inflammation, wound healing, and cell death [20,24].

Cancers, rather the cancer, are complex diseases. Apart from occupational cancers, cancer etiology is still largely unknown. From the mechanistic point of view, the “multistep” theory tries to rebuild the history of disease through three different stages. The first phase is initiation, where the cell shows an alteration of DNA; this phase may direct towards a second phase named promotion, characterized by an uncontrolled proliferation, after which the latent period is concluded, that in the human being was established lasting approximately 20 years. The third phase, named progression, completely shows the malignity of tumor by means of invasion and metastasis, two different processes that always result in the subject’s death.

While in the past the attention of scientists pointed to either genes or epigenetic factors, currently it appears much more important to evaluate the role played by the context where cancer cell lives, above all through its interaction with other host cells and ECM, then focusing on the microenvironment (ME) and last but not least the Immune System (IS) response that tries to overcome the uncontrolled cell proliferation and tissue invasion. We might name all these interactions “metagenetic factors” using an ancient Greek prefix that means “beyond”. In this regard, we verified the scientific literature, particularly focusing on the GJIC between cancer cells and endothelial cells (ECs), such communication allowing cancer cells to use ECs to achieve two different useful results: (1) Gaining needed nutrients by the blood supply in order to thrive; and (2) obtaining a possible way out from the tissue where tumor cells arise, the latter permitting cancer cells to colonize other organs during the metastatization process. In addition, ECs and cancer cells interact with the IS cells, and in this respect, such communication appears decisive to determine cell fate as the evolution or worsening of the disease depends on such interplay. In this context, cytokines play an essential role as capable of shaping an inflammatory or alternatively an anti-inflammatory state. These molecules produced by IS cells, but not just from them, orchestrate the immune cells and fibroblasts and contribute to the tumor ME (TME) where the cancer develops. The features of this milieu are crucial, configuring two possible mutually exclusive scenarios; one will enable cancer growth, another will contrast it, thus conditioning cancer fate. It is difficult to establish how much it depends on the interaction of different factors and how much they differently weight, but these processes certainly seem to depend on how the cells communicate to each other. Therefore, understanding exactly how it operates will be increasingly relevant to counteract cancer in a preventive/therapeutic context.

## 2. Search Strategy

The following search items, combined with the Boolean term “AND”, were used to perform an electronic search in the PubMed, EMBASE, and Scopus databases: Gap junctions, microenvironment, connexins, pannexins, metastasis, activating invasion, inflammasome, avoiding immune destruction. This is a narrative review of the literature and not a systematic review.

## 3. Activating Invasion and Metastasis

### 3.1. Role of GJIC and HCs in Cancer Cell Invasiveness

If we consider Cxs as tumor enhancers, it has been shown that Cxs were able to increase motility of the glioma cells due to GJIC with astrocytes, then promote intravasation and extravasation processes through mechanisms mediated by the Cx C-terminal domain, in a GJIC-independent-manner [25]. In addition, Panxs play a role in cell growth control, as well as in invasion and metastasis, even though their role is relatively unexplored weighed against Cxs [26].

In order to invade neighboring tissues, epithelial cancer cells have to overcome the basal membrane. For this result to be achieved, cancer cells need to communicate with other cells and with ECM. The first step is the disassembly of the adherens junction [27]. In this context, a fundamental role is played by TGF-β, in fact the inhibition of its receptor type-I decreases cancer invasiveness [28]. Moreover, TGF-β induces miR21, a main regulator of mesenchymal phenotype transition [29]. TGF-β also activates the mechanistic targets of rapamycin complex 1 (mTORC1) and mTORC2 [30]. Successively, TORC permits the translation of proteins fundamental for cell growth and development; the importance of the PI3K/mTORC1 pathway in cancer-associated inflammation has also been shown [31]. Another extracellular enzyme that appears important, Lysyl oxidase (LOX), is capable of enhancing the covalent crosslinking present in ECM fibers. In this context, we cannot omit matrix metalloproteinases (MMPs), in that both LOX and MMPs are also induced by TGF-β [32]. LOX also promotes the activation of PI3K [33]. In turn, AKT induces the phosphorylation of glycogen synthase kinase-3β (GSK3β) that stabilizes SNAIL [34], further increasing TGF-β-induced SNAIL [35]. In the same way, LOX acts by stabilizing SNAIL [36]. In addition, TGF-β promotes the dissociation of the long isoform of p120 from the membrane, then accumulating in the cytoplasm [37]. The net result is an activation of Cdc42—a cell-division controlling protein belonging to the family of Rho small guanosine triphosphatases (GTPases); contemporarily, an activation of Ras-related C3 botulinum toxin substrate 1 (Rac1) occurs, determining three different events: (a) Decreasing E-cadherin [38,39]; (b) microtubule polymerization [40]; and (c) integrin clustering [41]. Thereby, cell migration occurs, promoted through the destabilization of cell contacts with the basal membrane [42]. In addition, Rho activity due to binding to exchange factor Vav2 is suppressed by p120, in turn this event activates Rac1 [37]. Given that Rho GTPases regulates adherens junctions, the suppression of Rho destabilizes the adherens junctions, incrementing the dysregulation in the constitution of cell–cell complexes.

The events outlined above are an integral part of the epithelial-mesenchymal transition (EMT), currently considered a seminal process in cancer invasiveness and metastasis formation. Cx43 and GJIC are modulated during the EMT [43,44,45,46,47,48]. Hills and colleagues using renal proximal tubule cells provided evidence that the TGF-β1-promoted EMT events were associated with a loss of E-cadherin and cell adhesion, and ultimately, also Cx43-mediated cell communication [43]. James et al. [44] have recently shown that TGF-β1-mediated activation of ERK1/2 Smad3 led to suppression of Cx43 gene (*Gja1*) mRNA internal translation in mesenchymal cells and that this is sufficient to limit gap junction formation. Interestingly, it has also been shown that GJIC mediated by Cx43 did not affect the growth and migration of U2OS human osteosarcoma cells but suppressed TGF-β1-induced EMT only when U2OS human osteosarcoma cells were cultured with normal human osteoblasts [45]. Cancer-associated fibroblasts (CAFs) undergo increased aerobic glycolysis and promote EMT, migration, and invasion of non-small cell lung cancer (NSCLC) cells through the formation of unidirectional GJIC and metabolic coupling [49].

Once overcome the basal membrane, epithelial cancer cells migrate and come in proximity with tumor vessels. GIJC between circulating tumor cells and ECs was described by Ito et al. [50]. Other authors have reported evidence that Cxs could be fundamental in the communication between cancer cells and the ECs present in their cellular microenvironment; moreover, these interactions would modulate both processes of cancer cells migration and invasion [51]. Furthermore, since signaling molecules inciting angiogenesis, such as cytokines, are found in the TME, this interaction may represent an important link between inflammation and the local increment of primary tumors; in fact, the TME is where tumor cells, besides their intrinsic properties, are instructed to invade tissue and subsequently to metastasize.

### 3.2. Role of GJIC and HCs in the TME ATP/Adenosine Modulation

The role of extracellular adenosine triphosphate (ATP) concentration in the TME is highlighted by the observations that ATP is able to promote tumor growth [52] and angiogenesis [53] through P2R-mediated stimulation. ATP effects are finely regulated by its concentration, its linking to the P2 receptor (P2R) subtypes, and the expression levels of nucleotide degrading enzymes [54]. All these processes are viable in the TME [55,56,57,58] and they will be presented in the following paragraphs.

Beyond being an important source of energy in living cells, ATP is also a pleiotropic extracellular messenger, in fact it contributes to cell-to-cell communication by binding to plasma membrane receptors, particularly to P2 purinergic receptors [59]. There are two adenosine (P1) and nucleotide (P2) selective receptors. P1 receptors are further subdivided into A1, A2a, A2, and A3, whereas P2 receptors are subdivided into the P2Y and P2X subfamilies [60,61,62]. The affinity of P2 receptors for extracellular nucleotides is different, particularly as P2Y receptors are sensitive at the nanomolar level; instead, P2X7 is activated at micromolar levels. This wide range of affinities of P2 receptors along with their ubiquitous distribution in all tissues permit P2 receptors to constitute a signaling system very versatile and one of the most ubiquitous in the human body.

Different processes such as proliferation, differentiation, and migration show the involvement of extracellular ATP, ADP, and UTP. Moreover, they participate either as neurotransmitters or regulator of cytokine release, apoptosis, and necrosis [63]. ATP, when released in the extracellular environment, is able to regulate many physiological processes, including platelet aggregation, vascular tone, peripheral and central neurotransmission, cardiac function, and smooth muscle contraction [64]. On the other hand, ATP behaves as a danger signal for cells in other circumstances [65], despite it having a very short half-life following degradation by ecto-ATPase [66]. Finally, nucleotide signaling participates in important pathological events such IS maturation, neurodegeneration, inflammation, and cancer [67]. Then, nucleotides may be the ideal signal molecules to report cell damage or distress behaving as damage-associated molecular patterns (DAMPs), because they are more concentrated in the cytosol than in the extracellular space where they appear virtually absent [68].

In this context, the nucleotide-degrading system is relevant, since it contributes to generating adenosine (ADO), an additional powerful modulator of cell functions acting at P1 receptors [69]. The main enzymes involved in ADO generation are the ubiquitous ectonucleotidases CD39, which converts ATP to ADP and ADP to AMP, and CD73, which converts AMP to ADO. ADO plays a role as immunosuppressor, but it can either stimulate or inhibit tumor growth [58,70].

**Figure 2:**
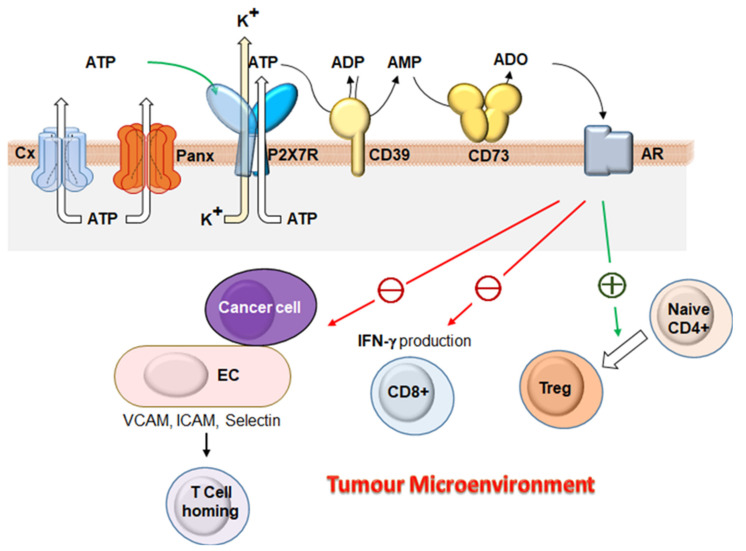
The Panx/purinergic system role in cancer facilitation. ATP is released into the extracellular space via different modalities, among which relevant to this review, Panx1-HCs, or P2XR7, amplifying the signal derived from ATP. P2XR7 is activated by extracellular ATP, that, in turn, activates P2X7R to trigger K+ efflux. Released ATP is hydrolyzed to ADP and AMP by ectonucleotidase such as CD39. AMP is further degraded to adenosine (ADO) by CD73 and activate adenosine receptors (AR). CD39 and CD73 are expressed not only by tumor stromal cells (such as endothelial cells or tumor-associated regulatory T cells) but also by certain cancer cells. Adenosine exerts its immunosuppressive effects by activating AR expressed by tumors cells, endothelial cells (EC), or immune cells, then acting in paracrine and autocrine way. Due to the activation of A2A adenosine receptors, an inhibition of IFN-γ production and cytotoxic killing by CD8+ T cells and promotion of CD4+ cells differentiation into T-regulatory cells occur. ADO also acts on the tumor-surrounding endothelium repressing T-cell homing to tumors via downmodulation of adhesion proteins such as ICAM-1, VCAM-1, or P-selectin on EC.

Therefore, the extracellular ATP concentration can change as a consequence of enhanced ATP release as well as of reduced ATP hydrolysis (Figure 2). During ischemia, hypoxia, inflammation, cancer, or trauma, an increase of the ATP extracellular concentration occurs, reaching the hundred micromolar level [71]. Thus, a number of candidate ATP-permeable release channels have been studied, e.g., voltage-dependent ATP-conductive large-conductance (VDACL) anion channel [72] or other chloride channels such as the cystic fibrosis transmembrane conductance channel regulator [73], ABC transporters [74], Cxs [75,76], Panxs [77], and the P2X7 receptor itself [78] (Figure 2). There is another method that cells use to release ATP: Via vesicular release, particularly used by mast cells, platelets, and neurons. Basically, this mechanism exists in each cell that displays stimulated or constitutive exocytosis [76]. Moreover, it is known that suffering or death cells release large amounts of ATP [79]. Recently, Michaud et al. showed that autophagy-competent cells were able to release ATP [80].

An important source of ATP is provided by mitochondria via its oxidative phosphorylation system. The ATP generated by the H+-FoF1 ATP-synthase inside the mitochondrial matrix is exchanged with cytosolic ADP via the adenine nucleotide carrier in a tightly controlled manner to guarantee the right refueling of energy to compartments outside mitochondria. Damaged mitochondria uncontrolledly release a number of molecules that function as pro-inflammatory signals collectively named mito-DAMPs or mitochondrial alarmins [81]. Mito-DAMPs have been identified as important pro-inflammatory mediators of the innate and adaptive by activating immune response by activating cell surface and intracellular receptors and implicated in various inflammatory and autoimmune conditions as well as in ischemic heart disease and cancer [82]. Mito-DAMPs comprise ATP as well as mtDNA, transcription factor A (TFAM), N-formyl peptydes (fMLP), succinate, cardiolipin, and cytochrome c [83].

Regarding the mechanisms of the unrestrained mitochondrial ATP release, this may occur via activation of the cyclofilin D-mediated mitochondrial permeability transition that when prolonged causes organelle swelling with release of pro-apoptotic factors [84]. Of note, it has been reported that connexin 43 also localizes in the inner mitochondrial membrane of cardiomyocites mitochondria where it assembles into hemichannels and is apparently implicated in the preconditioning protection of ischemic hearts [85]. Whether Cx-43 is present in mitochondria of other cell types has not been systematically investigated. Therefore, ATP can be released as mito-DAMP from mitochondria inside the cell contributing to paracrine/autocrine signaling or be discharged from apoptotic or necrotic cells. In addition, the presence of functional cell-free mitochondria in the blood has been recently unveiled as well as their secretion from tumor cultured cells [86]. Further studies are needed to investigate the role of mitochondria as signaling organelle outside the cell and whether these circulating units are involved in the development of human diseases.

ATP inevitably appears extracellularly in close vicinity to the site of tissue damage or inflammation. Nowadays, it is known that along with cytolytic liberation from damaged or dying cells, ATP may be released via non-cytolytic mechanisms from many cell types acting as an autocrine and/or paracrine signaling molecule by elevating the cytosolic Ca2+ concentration due to activating the ionotropic P2X receptors and metabotropic P2Y receptors present on the cell surface. ATP-induced purinergic signaling can significantly influence stem and progenitor cell behavior, in particular mesenchymal stem cell (MSC) migration [87]. Bone marrow (BM)-MSCs, in turn, are able to release ATP following stimuli such as fluid flow-induced shear stress [88] or shockwaves [89]. When in the TME, MSCs display many pro-tumoral activities, including the fostering of tumor growth and the stimulation of EMT, thereby promoting cell motility, invasiveness, and metastasis [90].

Cx HCs are involved in mediating constitutive ATP release from BM-MSCs, as shown by blockers of the Cx-HCs such as octanol, palmitoleic acid, or 18-α-glycyrrhetinic acid [91]. Different authors showed that extracellular ATP was capable of inducing cell migration, and these observations were reported regarding either epithelial cells or microglial cells [92,93,94]. Particularly, extracellular ATP regulated the migration of cancer cells favoring cancer invasion or metastasis [95]. In this respect, the role of P2X7 [96,97,98,99] and P2Y2 receptors [100,101,102,103,104,105,106] appears crucial, because they were shown to mediate the ATP-induced regulation of cancer cell migration.

Panxs are also critical players in ATP release related to both acute inflammation and cell death [20]. The mechanism of Panx activation is quite complex. In keeping with its role in inflammatory ATP release, it has been found that Panx1 can be activated by both irreversible caspase-mediated cleavage [107] and reversible G-protein-coupled receptor (including α1-adrenoceptor-mediated) pathways [108]. It has been proposed that physiological and pathological roles of Panx1 depend on its open state probability, which in turn is influenced by the increase in intracellular Ca^2+^ and extracellular ATP and K^+^. Additionally, Panx1 activation is regulated by its interactions with purinergic P2 (e.g., P2X and P2Y) receptors, which are activated by binding extracellular ATP at the plasma membrane [109]. Panx HCs, and downstream P2 receptors may act releasing low or high ATP levels. Low (normal) ATP, released by Panx1 HCs, is required for homeostatic cell functions (regenerative growth, proliferation, migration). On the other hand, massive (pathological) extracellular ATP leads to sustained activation of Panx1/P2X signalosome and subsequent prolonged inflammation and pyroptotic death.

**Figure 3:**
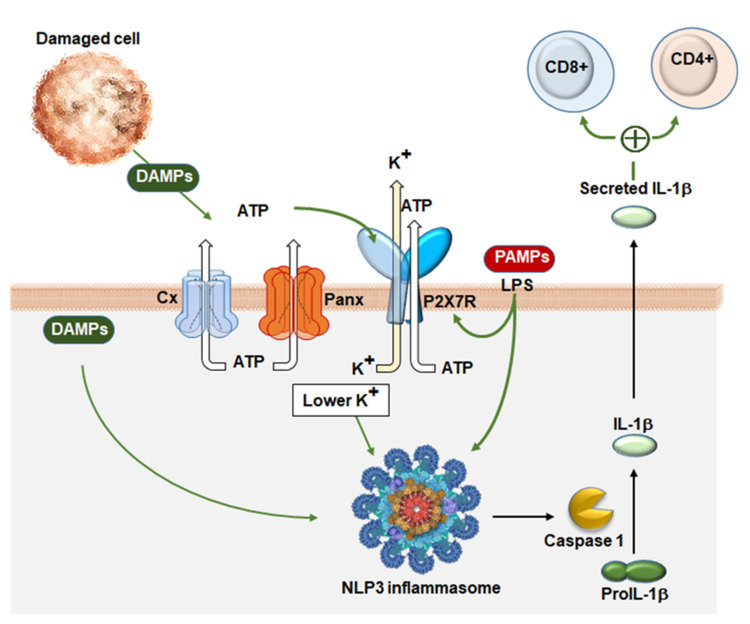
The Panx1/PR signalosome in anti-cancer innate immunity. When a pathogen-associated molecular pattern (PAMP), such as lipopolysaccharide (LPS), binds to innate immunity receptors of phagocytic and dendritic cells (e.g., toll-like receptors, not shown), the activation of the NF-κB signaling cascade ensues (not shown), which upregulates the expression of the inflammasome component NLRP3, pro-IL-1β and TNF-α, and the whole complex associates with P2X7R. DAMPs, either shed from dying cancer cells and acting at the plasma membrane or in the cytosol, including ATP, and PAMPs, may cause Panx1 opening and ATP release, which activates P2X7R, allowing calcium flow into the cells and K+ efflux. Lower intracellular K^+^ provokes inflammasome assembly, bringing NLRP3 and pro-caspase 1 together with the adaptor protein ASC. The inflammasome activates pro-caspase 1, that will cleave the pro-peptide from IL-1β, which is then secreted from the cell along with TNF-α (not shown). Both cytokines increase CD4+ and CD8+ T cells, mediating antitumor responses.

Due to the link between inflammation and cancer, it is essential to report that Panx1 activated the inflammasome in many cell types (Figure 3), as macrophages [110,111], microglia [112], neurons, and astrocytes [113]. Particularly either inflammasome assembly and processing of Casp1/11, IL-1β, and IL-18 precursors are regulated by Panx1/P2X signalosome, to facilitate ATP and K^+^ release, as well as uptake of extracellular Ca^2+^ and danger/pathogen-signaling patterns [114,115]. Recent data show that Panx1 is only required for NLRP3 inflammasome assembly during apoptosis but is dispensable for canonical NLRP3 or noncanonical inflammasome activation in BM-derived macrophages [116].

A truncated form of the Panx1, PANX1 (1–89), was found recurrently enriched in highly metastatic breast cancer cells, allowing metastatic cell survival during traumatic deformation in the microvasculature by augmenting ATP release [117].

In order to foresee new cancer treatments, it might be useful to identify novel therapeutic agents able to induce a different equilibrium between ATP release and ATP degradation, with the caveat that the two options are necessarily mutually exclusive, as they correlate specifically with invasiveness (ATP degradation) or corrected immune response to the cancer growth (ATP release).

ATP release through Panx1 HCs and the role of P2X receptors and particularly the P2X7R receptor in the TME appear seminal, since they may determine two opposite effects: Cell growth or apoptosis. In a transient activation state, P2X7R behaves as cationic-selective channels and might induce cancer cell growth [118,119], whereas its sustained activation promotes a characteristic channel-to-pore transition that drives formation of a non-selective pore, leading to cell death [120,121]. P2X7R was found to cause also NLRP3 inflammasome activation, IL-1β release, and cell proliferation [118,119,121,122] (Figure 3). Adinolfi et al. showed that transfection of P2X7R into human HEK-293 fibroblasts or mouse CT-26 colon carcinoma cell lines, enhances tumor engraftment. This procedure was able to stimulate not only in vivo either growth rate and the rate of proliferation, but also angiogenesis, and contemporarily reduced apoptosis [123]. In an in vivo model of neuroblastoma, it has been shown that ATP TME content increased in parallel with tumor progression and likely contributed to myeloid-derived suppressor cells [124]. Extracellular ATP, released in TME by cancer cells and infiltrating inflammatory cells, interacts with P2X7R expressed by DCs to stimulate IL-1β release, which in turn increase CD4+ and CD8+ T cells, mediating antitumor responses [125].

Collectively, all the available data indicate that Panxs and HCs are fundamental to decide if tumor may invade neighboring tissue and then to migrate, however other studies are needed to explain the complex interaction between molecules as cytokines, ATP, ADO, and endothelial, epithelial, and immune cells.

All these observations are the seeding for further issues. A first issue would be whether the cells are able to modify TME via HCs, on the other hand we should consider that the cells themselves may react at this change using cell-cell communication via GJs. It follows that GJIC might facilitate or contrast TME modification, so that invasiveness/metastatization might be enhanced or stopped. A second issue arises: If cancer derives by a lack of a positive feedback due to GJIC, this would indicate the relevance of GJs, and in this context, an inhibition of GJIC might be the cause of cancer progression. A third question is the following: If GJIC are able to spread DAMPs [126], an inhibition of GJIC would augment factors enhancing tumor migratory behavior and invasiveness, while functioning GJs would dilute them, thereby preventing the onset and progression of cancer.

## 4. Avoiding Immune Destruction

The immune surveillance theory of tumors was proposed more than 60 years ago by Burnet and Thomas [127]. According to it, the IS explores the microenvironment eventually identifying cancer-initiated cells that are recognized as non-self and trying to get rid of them. The elimination of cancer cells is orchestrated by both innate and adaptive immune cells, namely APCs, constituted by various subsets of T cells, B cells, and NK cells [128]. Nowadays, cancer cells have been recognized to be able to escape from the IS in keeping with the so-called immunoediting theory. As proposed by Dunn and Schreiber [129], three essential phases characterize the interactions between cancer and IS cells: Elimination by NK, CD4+, and CD8+ T cells, equilibrium, and escape from cancer immune surveillance.

As outlined above, the purinergic system, in its interplay with Panx HCs, can regulate tumor growth and angiogenesis in the TME. In general, ATP can be conceived as a “find me” signal that promotes the immune recognition by attracting effector cells. On the other hand, ATP is converted to ADO by the action of CD39 and CD73 ectonucleotidase pathway. ADO directs the phenotype of recruited immune cells in the TME and, by this action, functions as an immunosuppressive agent [57,58,130,131] (Figure 2).

It is well known that the hypoxic state of the center of a tumor mass induces hypoxia inducible factor 1α/β (HIF-1α/β), that in turn incites the expression of CD39 and CD73, thereby ADO formation is promoted [132,133,134,135]. The relevance of CD39 and CD73 in modulating ATP and ADO levels in the TME also comes from the observation that cancer cell lines, immune cells, and stromal cells express these enzymes, and then they may be considered immune checkpoints in cancer [130]. It has been shown that T cells are inhibited by tumor-produced extracellular ADO because of the A2AR-triggered elevation of intracellular levels of cAMP and subsequent TCR signaling and IFN-γ production [136]. ADO has multiple inhibitory effects on the IS orchestrating the TME to promote tumor growth [137], as shown in Figure 2. AR activation by ADO inhibits IFN-γ production by T cells [138] and the activation of CD8+ T cells [139]. ADO has been shown to increase the numbers of Tregs and further promote their immunoregulatory activity [140]. Besides these direct effects on T cells, ADO has also an indirect effect by diminishing adhesion molecules on EC of the TME (VCAM, ICAM, selectin) inhibiting T cells extravasation [141,142].

Consistently, supplemental oxygenation (hyperoxia) was found to decrease intratumoral hypoxia and the concentrations of extracellular ADO in the TME [143], induce in vivo tumor regression, increase activities of anti-tumoral T and NK cells, reduce immunosuppression executed by regulatory Tregs, and increase levels of pro-inflammatory cytokines and chemokines. Hyperoxia worked at the root of immunosuppression in the TME, i.e., by reversing ADO effects [134]. Additional studies have shown that the deletion of A2AR and its signaling lead to an enhancement of anti-tumor activities of T cells [144], as well as NK cell maturation is promoted with anti-tumor effects [145].

We might question whether cancer cells are the only culprit in the orchestration of immunosuppressive actions of ADO. Indeed, it has been proposed that the hypoxia–HIF-1α–adenosine–A2AR signaling pathway can be amplified by influencing myeloid cell components of the TME. These include tumor-associated macrophages (TAMs) and myeloid-derived suppressor cells (MDSCs), since it has been shown that hypoxia and HIF-1α drive their recruitment to the TME, as well as M2-like polarization and activity [146,147,148]. ADO-generating ovarian cancer cells attract in vitro differentiated TAM-like cells, which in turn upregulated the expression of CD39 and CD73 and suppressed CD4+ T cell proliferation. Since stromal fibroblasts (SFs) also express CD73 in vivo, it is conceivable to hypothesize a collaboration of TAMs and SFs in amplifying ADO generation and hence the immunosuppressive effect [149].

Hypoxia in the TME alters the IS activity by increasing the resistance of tumor cells to NK cells [150,151] and to cytotoxic T-cells [152,153] via the induction of HIF-1α [154]. Tittarelli et al. [155] showed in melanoma cells that despite Cx43 protein expression was determining the formation of functional Cx43-HCs, likely stabilizing the immunological synapse, hypoxic cells were less susceptible to NK-mediated lysis than normoxic cells. They also established that autophagy occurring in hypoxic melanoma cells caused the selective degradation of Cx43-GJIC impairing the immune synapse between melanoma cells and NK cells.

In conclusion, ATP can be secreted in tumor interstitium practically by all cell types present at the tumor-host interface. Extracellular ATP might act as a “find me” signal attracting and activating immune cells in the TME. However, this response seems to be subverted by ADO generation through the ectonucleotidase pathways. Another way by which ATP/ADO ratio can determine immune subversion is via the recruitment of MDSCs. These MDSCs are comprising immunosuppressive cell subsets isolated from neuroblastoma (NB)-bearing mice. In this tumor, it was shown that MDSCs are able to suppress adaptive and innate immunity [156] by different mechanisms including above all inhibition of antigen-specific and non-specific T-cell activation [157,158], induction of M2 macrophages that contribute to tumor progression and invasion [159,160], stimulation of Treg expansion [161,162], and repression of NK cytotoxicity [163].

By studying P2X7R expression and function in either granulocytic or monocytic MDSCs (G- or M-MDSCs) in NB, Bianchi et al. [164] found that plasma membrane expression of P2X7R in M-MDSCs was coupled to increased functionality, following upregulation of ARG-1, TGF-β1, and ROS, i.e., important immunosuppressive factors, and production of the chemokine CCL2 [124]. Moreover, ADO can be generated by G-MDSCs, expressing high levels of CD73, contributing to MDSC expansion and immunosuppressive activities through ligation of A2B receptors. On this basis, a scenario has been proposed that highlights that both autocrine and paracrine loops are at work: Extracellular ATP released by tumor cells interacts with P2X7R expressed by the same cells or adjacent tumor cells and drives tumor cell proliferation [165]. As an alternative, ATP secreted by NB cells interacts with P2X7R expressed by MDSCs and stimulates the release of CCL2, involved in TAMs and iNKT recruitment. On the other hand, ADO generated by G-MDSCs contributes to MDSC expansion and immunosuppressive activities through A2B receptors. Finally, M-DSCs from tumor-bearing mice express P2X7R, which increase tumor cell proliferation mediated by substance P secretion. [165].

In conclusion, the tumor growth might be modulated by acting on purine receptors and enzymes that hydrolize ATP in ADO, even though a receptorial heterogeneity exists in tumors. In addition, targeting P2X7R might result in being useful in the reduction of immune suppression and in the induction of a protective immune response against cancer cells.

### 4.1. Immune Evasion and Cell Deaths: Therapeutic Strategies

Here we would remark that tumors are able to escape immune eradication by dysregulating the balance between the effector and regulatory cell compartments [166]. A possible therapeutic strategy was used inducing a type of cancer cell death capable of stimulating immune response. In this context, it appears useful to observe that the different forms of cell death play an important role: In order to better explain this scenario, we note that after apoptosis, cellular debris are recognized by macrophages and dendritic cells [167], without inducing immunological responses because of the release of anti-inflammatory signals [168]. Another type of death that may occur is autophagy, appearing during starvation and used by cancer cells when they do not receive nutrients or oxygen. Thus, autophagy might help cancer progression. The third type of death is necrosis, characterized by the massive release of pro-inflammatory mediators, that induces an important immunological response. Because of the release of DMAPs due to the membrane rupture, this process is also named necroinflammation [169]. Usually, cancer cells are poorly immunogenic generating anti-inflammatory cytokines [170,171]. In fact, because apoptosis is tolerogenic, an anticancer strategy is to stimulate immune response by a set of therapeutics able to cause another type of apoptosis named immunogenic cell death (ICD) [172]. ICD is elicited by DAMPs released by dying tumor cells resulting in a T-cell-mediated activation against cancer.

It is interesting to note that specific treatments that increase the immunogenicity of cancer cells may enhance long-term antitumor immunity [173]. Certain chemotherapeutic agents can induce antitumor immune responses through their ability to trigger ATP release by tumor cells [79,125]. It has also been hypothesized that anti-cancer treatments stimulate anti-neoplastic immunity through DC maturation, cytotoxic T lymphocytes’ activation by activating ICD [174], that also increases the cytotoxic function of NK cells [175]. In particular, these immunostimulatory effects are exerted by DAMPs [172]. DAMPs may be present on cell surface (i.e., Calreticulin (CRT), Heat Shock Protein (HSP 70–90)), or in the extracellular space ((High Mobility Group Box 1 (HMGB1), uric acid, pro-inflammatory cytokines, ATP, DNA, and RNA) [166,176,177]. DAMPs induce phagocytosis and activation of T lymphocytes to eradicate tumor cells [178].

In regard to CRT, some studies report its role in the immunogenicity of dying cells, functioning as an “eat me signal” when exposed on the cell surface and activating APCs [179,180]. CRT is secreted upon the activation of the unfolded protein response occurring in the endoplasmic reticulum (ER) stress determined by ICD inducers.

ATP is another DAMP crucial for effective elicitation of ICD [7,9,66]. ATP release occurs following ICD inducers through the action of a lysosomal-associated membrane protein (LAMP) 1 dependent mechanism. LAMP1 translocates to the cell surface in a caspase- and Panx1-mediated process [181]. Extracellular ATP facilitates strong chemotactic effects by binding to purinergic receptors P2RY2 and P2RX7 on APCs and their precursors, respectively [125,182]. So, the dying cell’s immunogenicity is abolished not only when ATP fails to gain access to the extracellular milieu of these cells [182,183], but also when P2RY2 or P2RX7 are absent from the myeloid cells. As outlined above, P2RX7 signaling activates the NLRP3 inflammasome, which in turn induces the secretion of active IL-1β, this cytokine playing an important role in the amplification of an efficacious anti-cancer immunity [125]. Furthermore, secreted ATP is able to amplify the motility of DCs [184] and tumor cells [185]. Finally, the maturation of DCs and the expression of the co-stimulatory molecules can be supported by ATP [73]. The apoptotic phase of cell death that stimulates ICD can be important in the secretion of ATP [8].

In this scenario, another important player is HMGB1, a nonhistone nuclear protein. When HMGB1 localizes in the extracellular environment, it acts as danger signal informing adjacent cells, causing inflammation, and inducing immunity by binding different receptors [87,88,89]. An active secretion of HMGB1 also exists, and in both cases, binding to TLR4 on immature DCs is able to stimulate a myeloid differentiation primary response that determines a DC maturation and activation of a cytotoxic T cells’ response [90,91]. Heat shock proteins (HSP) and IFN are also powerful immunostimulators, with the latter acting either stimulating antigen presentation or promoting antitumor T cells.

Particularly intriguing seems to be the different mechanisms used by chemical and physical anti-tumoral agents such as ICD inducers. In fact, while for example the chemotherapeutic drugs act by provoking, above all, apoptosis and indirectly ER stress, physical ICD-inducers would directly induce ER stress. Of particular importance is the role of anthracycline because anthracycline-driven ICD seems capable of inducing the infiltration of IL-17-producing γδ T lymphocytes and subsequent invasion of CTLs into TME. Indeed, the therapeutic effects of anthracyclines were abrogated following the suppression of γδ Th17 cells by the knockout of T cell receptor δ with the consequence of inhibition of the production of IL-17 by T cells in the TME. Accordingly, inhibition of the IL-17A–IL-17R pathway led to a reduced tumor-specific T cell response, substantiating an essential role for γδ T17 cells in anthracycline-induced immunity [186]. Considering physical anti-tumoral agents, High Hydrostatic Pressure (HHP) was capable of inducing the same effect of anthracycline via ATP release into extracellular environment and phagocytosis of tumor cells [187]. Cancer-specific immune reactions using HHP treatment to generate a DC-based anticancer vaccine were the focus of different clinical trials for ovarian and prostate cancers [188]. Using HPP, killing tumor cells seems a task easily performed, with the aim to produce a standardized protocol for cancer immunotherapy [189].

The modern photodynamic therapy (PDT) is a result of the combination of light and chemicals for better therapeutic efficiency [190], starting a chain reaction leading to the production of ROS, then determining cell death. Moreover, the activation of IS by PDT is testified by the recruitment of inflammatory cells such as macrophages, leukocytes, and lymphocytes into the TME [191,192]. PDT-mediated upregulation of pro-inflammatory cytokines, IL-6, and IL-1, but not TNF-α, has been reported [193]. Interestingly, PDT is able to induce ICD [194], with the result of CRT, HSP70, and HSP90 translocation from ER to the plasma membrane and extracellular secretion of HMGB1 and ATP [195,196].

In conclusion, these strategies induce an immune response as therapeutic ally, avoiding apoptosis that is normally tolerogenic and thus incapable of alerting the body.

### 4.2. The Role of Cytokines and GJIC in the TME

The role of cytokines cannot be omitted in the crosstalk between stromal cells and cancer cells, because as noted above, cancer cells derive their fate by the TME. If TME may favor or contrast the cancer growth, then the role of cytokines produced by immune cells, although not only by them, has to be closely examined.

Considering inflammation at early stages of cancer, cytokines have been shown to downregulate GJIC. For example, polycyclic aromatic hydrocarbons (PAHs) exposure of mouse lung epithelial cells negatively influenced GJIC and this effect was mediated by TNF produced both by epithelial cells and macrophages [197]. A role for heterologous GJIC between malignant glioma cells and astrocytes via Cx43 GJs has been suggested in tumor cell adhesion and invasion [198,199]. The F98 glioma cells, although presenting functional GJs, did not show responsiveness to TNF-α treatment. However, intracellular application of TNF-α robustly inhibited functional coupling, likely due to activation of protein kinase C and Cx43 phosphorylation [200]. In addition, our research groups verified an interplay between GJIC and cytokines showing that mercury chloride reduced IL-1β and TNF-α in human keratinocytes cultures and that this effect was likely post-translational [201,202]. In this regard, if IL-1β causes inhibition of GJIC, negative feedback between the two pro-inflammatory cytokines and GJIC could exist, then a reduced GJIC would be able to reduce pro-inflammatory cytokines, and this loop might be important in the early phases of carcinogenesis.

A very important role to determine immune evasion by cancer cells is that played by interleukin (IL)-1α, which is released by many cell types in the response to necrotic cell death [203], for example, following hypoxia [204]. In this regard, IL-1α released by necrotic hepatocytes was able to induce carcinogenesis [204]. There are two IL-1α forms: One released upon inflammasome activation [205], and a membrane-bound form, that is able to activate IL-1 receptors on target cells such as T-cells and endothelial cells as well as to stimulate induction of other cytokines [206]. While the secreted form of IL-1α is highly pro-inflammatory in the TME and involved in tumor growth and invasiveness, its membrane form promotes anti-tumor immunity, and induces a reduction of tumor growth and invasiveness [206].

Necrotic cells and different tissues under stress conditions also release IL-33 [207]. Tumor tissue shows increased endogenous IL-33 expression, contributing to cancer progression [208,209,210]. The crosstalk between tumor cells and surrounding stroma is proved by the occurrence that IL-33 upregulation in tumor correlates with increased expression of target receptor complex IL-1 receptor-like 1 in stromal cells [209,210], indicating a paracrine effect of IL-33.

Limoge et al. [211] have shown in breast tumors that cancer cells were able to stimulate TME cells to produce cytokines such as transforming growth factor-β (TGF-β) and pro-inflammatory cytokines like TNF and IL-1β. These cytokines cooperate in activation of the MAPK-AP1 (JUN/JUNB) pathway, which, acting together with the TAK1-RELA axis, up-regulates expression of MMP9. This metalloproteinase, in turn, stimulates tumor neo-vascularisation by releasing and/or activating matrix-deposited pro-angiogenic growth factors, such as VEGF, thereby recruiting endothelial cells [212] and pericytes [213,214].

Elisha et al. [215] have demonstrated that the cooperativity between stromal cytokines drives the invasive migration of human breast cancer cells, transforming a TME capable to contrast cancer into one that enhances cancer. After having considered that both IL-1 and IL-6 have to be present for induction of invasive migration, these authors underline the effect of IL-1 on NF-κB and IL-6 on STAT3, then an important loop would develop that increases each other. HS5, a stromal cell line that originates from the bone marrow and is able to secrete many compounds including IL-1 and IL-6, induced another cell line, CCD1069, that normally blocks stromal invasion to produce IL-6, converting these cells from “invasion blockers” to “invasion promoters” [215].

In the opinion of Enns et al. [216], a likely link between inflammatory activation and cancer is hypoxia. In fact, hypoxia selects tumor cells with increased invasiveness and stabilized H1F-1α, a transcription factor capable of regulating genes coding for pro-tumor cytokines that act on stromal cells such as macrophages and fibroblasts to support an invasive tumor cell phenotype. Contemporarily, HIF-1α is involved in the switching of tumor cells’ energy production from OXPHOS to glycolysis, enhancing invasive qualities of tumor cells. In this regard, ROS increment would not be a consequence of cellular damage, but an active process that would favor cancer cells.

The relationship between inflammation and cancer in brain tumorigenesis has been thoroughly reviewed by Mostafa and colleagues [217], which affirm that instead, to protect the body, as it could occur through a well-regulated inflammatory response, “ironically chronic inflammation leads in the opposite direction”. They hypothesize two different oncogenic pathways, one starting from genes (intrinsic pathway), the other acting as a “driving force” enhancing cancer development (extrinsic pathway). Their focus is on chronic inflammation where the cells secrete higher levels of immune inhibitory cytokines as IL-6 and TGF-β able to degrade matrix promoting cancer invasion, instead of cleaning up cell debris and unwanted cells as they would accomplish during acute inflammation. In this regard, it is worth remembering the role of macrophages, particularly the alternatively activated M2 macrophages, capable of secreting immunosuppressive cytokines as IL-10 and TGF-β. Among cytokines, the relevance of IL-6 is testified by the fact that, when produced by astrocytes, it facilitated induction of angiogenesis, cell proliferation, and resistance to apoptosis [218]. In addition, IL-1β contributes to tumor growth and metastasis in different brain tumors [219]. In glioma TME, TNF-α promotes tumor formation and angiogenesis, and induces neovascularization through VEGF and IL-8. Contemporarily, TNF-α appears to play a fundamental role enabling glioma cells to escape from immune response allowing the appearance of an aggressive growth phenotype in the inflammatory TME. TGF-β activates the transcription factors SMADs (mothers against decapentaplegic homolog), that in turn stimulate different genes involved in the angiogenesis and migration/invasion as those encoding VEGF [220]. Moreover, it exerts an important immunosuppressor effect on infiltrating T-cells [221]. The anti-inflammatory cytokine IL-10 has been shown to induce a skewing from a Th1 immune-phenotype (with anti-tumor activity) to a predominant Th2 response (with pro-tumor activity) [222]. Finally, it appears important to underline the cooperative action of IL-6, IL-10, and TGF-β in determining higher STAT3 activity and thus STAT3-mediated negative effects on the immune responses in the tumor stroma [223]. Conclusively, the authors suggest a link between chronic inflammation and increment of ROS, the latter promoting genetic and epigenetic alterations followed by microsatellite and chromosomal instability. ROS could directly or indirectly affect DNA repair machinery and cell cycle checkpoints as well, thereby precipitating genomic diversification and intra- and inter-tumoral heterogeneity in brain cancer.

The conclusion of this loop is intriguing although appointed only on genes, but the cancer is not just a genetic disease and, in our opinion, all cannot end here. We instead hypothesize another explanation to bring these observations together. As a first consideration, we should initiate to modify the thoughts always derived by simple and direct causal connections. In this context, we would like to consider, for example, that in the interplay between TME and cancer cells, there is not a simple relationship, but we have to include the connections that each cell has with neighboring cells through GJs. Secondly, further studies are needed to explore molecular mechanisms acting in the TME, as well as the role exerted by various cell types, such as different kinds of immune cell, fibroblasts, and endothelial cells. Each of these is able to produce molecules and their crosstalk is more important than any single molecule that we are observing in a given moment. In this regard, the modulation of different cytokines that participate in the crosstalk might be a useful strategy as verified by Qing-An Jia et al. [224].

Overall, we should verify the effect of cytokines on GJIC. In fact, in early cancer stages, inhibition of GJIC appears to be carcinogenetic, while in late stages, functioning GJs appear to be enhancing cancer progression and invasiveness [25]. Similarly, in early stages, anti-inflammatory cytokines appear essential to induce immunosuppression; instead, pro-inflammatory cytokines would contrast cancer, and conversely, in late stages, pro-inflammatory cytokines play a crucial role to facilitate cancer progression also providing new allies to cancer cells as CAFs or M2 macrophages.

## 5. Conclusions

The formation of HCs and GJs, these last operating GJIC among cancer cells and HCs among cancer cells and TME cells, plays extremely complex and contrasting roles in tumor onset and progression. In this review, we have focused our attention essentially on the interplay between Cx and Panx-HCs and the purinergic system. It seems that above all, the Panx1-HC/P2X7R signalosome may either induce the tumor growth and invasiveness by increasing extracellular ATP levels or suppress the IS by the conversion of ATP into ADO. In this light, the ATP/ADO ratio is essentially the result of ATP-releasing channels and/or transporters and ectonucleotidases, which could be different from tumor to tumor in their expression and function. It will be essential to better study all these interactions and use this knowledge to attain more personalized and precise medicines for each tumor type and likely for each subject bearing a specific tumor.

Let us say that the methods adopted to contrast cancer by using therapeutic arms appear to follow a war strategy: Fight the enemy and transform enemies into allies in order to increase defensive arms. As men during the war use cryptic communication, transform language, and inhibit or sabotage the media, so too does the body, in a natural way, blocking the tumors in the early stage. When it fails, we should understand why, then project chemopreventive actions or other weapons, and draw action plans to promote general preventive arms such as the correct diet, regular moderate physical exercise, avoiding drugs and alcohol, adopting preventive measures at work, and eluding psychological stress through appropriate coping strategy. When the cancer is just developed, we should understand better which are our allies and which are our enemies and above all we should remember that an ally can become an enemy. In this case, an individualized therapy following deeper knowledge of a patient’s disease can make a difference.

In this regard, we could conclude remarking that during early stages of cancerogenesis enhancing GJIC prevents cancer and inhibiting GJIC promotes it. Instead, during the later stages, the opposite occurs. HCs would act as body allies through ATP release signaling a threat, whereas stromal cells expressing degrading enzymes (CD39 and CD73) are capable of paralyzing the IS. Two different therapeutic arms could be used, inhibiting these enzymes, or acting on purine receptors that modulate the immune response; in this context, cytokines and other communication strategies that cells use appear in the background. The use of cytokines as therapeutic weapons is even harder, due to systemic effects that are different in several individuals. Moreover, a preventive strategy could be increasing Th1-mediated defense and avoiding an imbalance toward the Th2 arm. Considering the literature, a preventive strategy would be essential in early stages of cancer by using anti-inflammatory drugs and immunomodulators strengthening body defenses; vice versa, during late stages, promoting a correct inflammatory response capable of removing the invader could be a resolutive strategy.
